# The genome of the emerging barley pathogen *Ramularia collo*-*cygni*

**DOI:** 10.1186/s12864-016-2928-3

**Published:** 2016-08-09

**Authors:** Graham R. D. McGrann, Ambrose Andongabo, Elisabet Sjökvist, Urmi Trivedi, Francois Dussart, Maciej Kaczmarek, Ashleigh Mackenzie, James M. Fountaine, Jeanette M. G. Taylor, Linda J. Paterson, Kalina Gorniak, Fiona Burnett, Kostya Kanyuka, Kim E. Hammond-Kosack, Jason J. Rudd, Mark Blaxter, Neil D. Havis

**Affiliations:** 1 Crop Protection Team, Crop and Soil Systems Group, SRUC, West Mains Road, Edinburgh, EH9 3JG UK; 2 Department of Computational and Systems Biology, Rothamsted Research, Harpenden, Hertfordshire AL5 2JQ UK; 3 Department of Plant Biology and Crop Science, Rothamsted Research, Harpenden, Hertfordshire AL5 2JQ UK; 4 Institute of Evolutionary Biology, School of Biological Sciences, University of Edinburgh, Edinburgh, EH9 3TF UK; 5 Edinburgh Genomics, The University of Edinburgh, Edinburgh, EH9 3JT UK; 6 Present address: Forest Research, Alice Holt Lodge, Farnham, Surrey GU10 4LH UK; 7 Present address: Syngenta, Jealott’s Hill International Research Centre, Bracknell, Berkshire RG42 6EY UK

**Keywords:** Ramularia leaf spot, Dothideomycetes, Rubellin toxin, Endophyte, Necrotroph, Whole genome sequencing

## Abstract

**Background:**

*Ramularia collo*-*cygni* is a newly important, foliar fungal pathogen of barley that causes the disease Ramularia leaf spot. The fungus exhibits a prolonged endophytic growth stage before switching life habit to become an aggressive, necrotrophic pathogen that causes significant losses to green leaf area and hence grain yield and quality.

**Results:**

The *R. collo*-*cygni* genome was sequenced using a combination of Illumina and Roche 454 technologies. The draft assembly of 30.3 Mb contained 11,617 predicted gene models. Our phylogenomic analysis confirmed the classification of this ascomycete fungus within the family Mycosphaerellaceae, order Capnodiales of the class Dothideomycetes. A predicted secretome comprising 1053 proteins included redox-related enzymes and carbohydrate-modifying enzymes and proteases. The relative paucity of plant cell wall degrading enzyme genes may be associated with the stealth pathogenesis characteristic of plant pathogens from the Mycosphaerellaceae. A large number of genes associated with secondary metabolite production, including homologs of toxin biosynthesis genes found in other Dothideomycete plant pathogens, were identified.

**Conclusions:**

The genome sequence of *R. collo*-*cygni* provides a framework for understanding the genetic basis of pathogenesis in this important emerging pathogen. The reduced complement of carbohydrate-degrading enzyme genes is likely to reflect a strategy to avoid detection by host defences during its prolonged asymptomatic growth. Of particular interest will be the analysis of *R. collo*-*cygni* gene expression during interactions with the host barley, to understand what triggers this fungus to switch from being a benign endophyte to an aggressive necrotroph.

**Electronic supplementary material:**

The online version of this article (doi:10.1186/s12864-016-2928-3) contains supplementary material, which is available to authorized users.

## Background

**Fig. 1:**
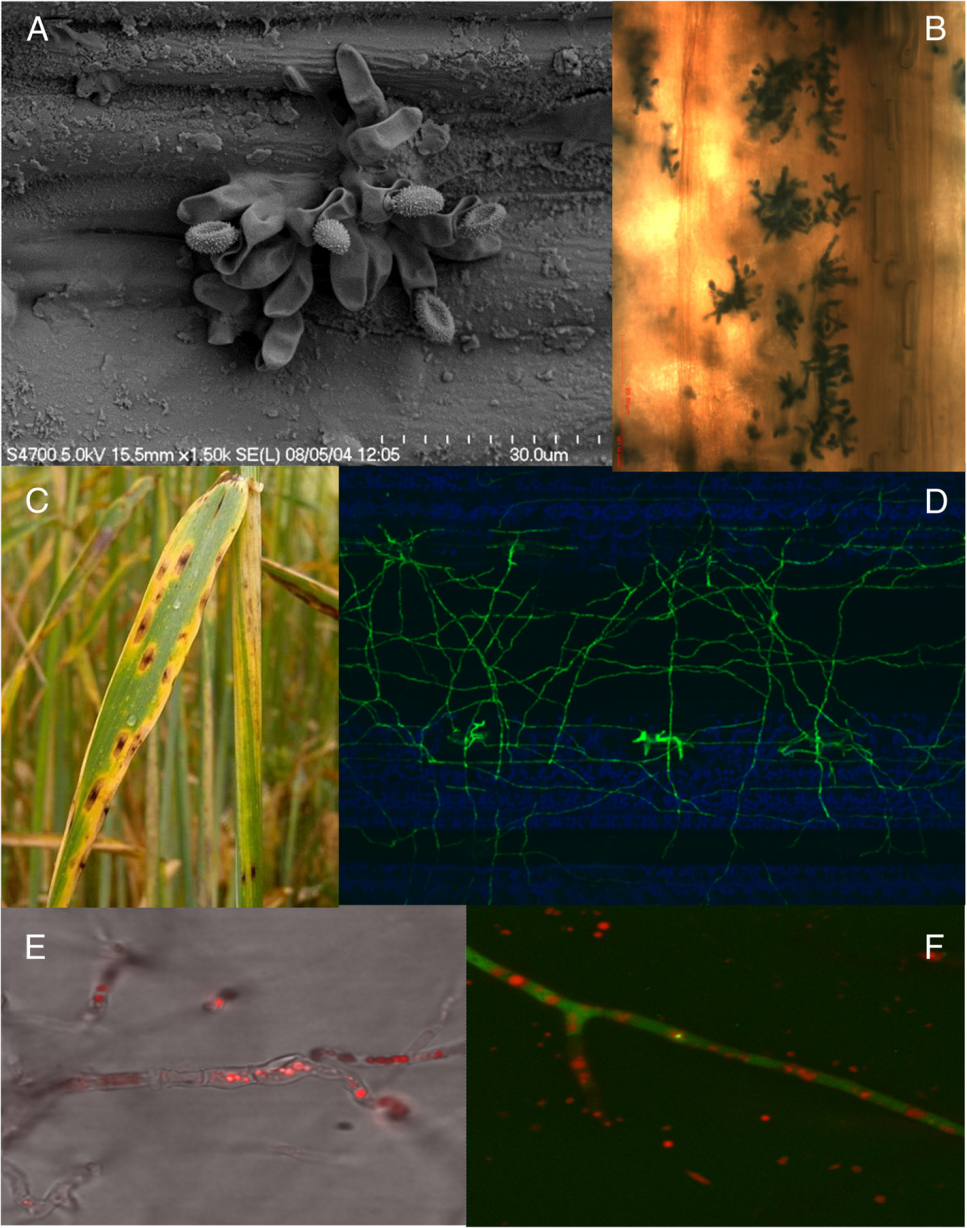
Ramularia leaf spot symptoms and *R. collo*-*cygni* development on barley. **a** Electron micrograph of the characteristic swan neck-shaped conidiophore of *Ramularia collo*-*cygni*. **b** Light micrograph of a cluster of *R. collo-cygni* conidiophores, from field infected plants, erupting from spring barley cv. Cocktail leaves at GS80+. **c** Characteristic Ramularia leaf spot symptoms on leaves of winter barley cv. Saffron. **d** Spider-web growth of GFP-transformed *R. collo*-*cygni* isolate Rcc-8B9-GFP [10] on leaf surface of spring barley cv. Optic, 7 days post infection without triggering host immune response. **e** Confocal image of rubellin autofluorescence in *R. collo-cygni* isolate B1, superimposed on bright field transmission image. Note various sizes of vacuole-like vesicles within the collapsed hyphae. **f** Confocal image of a viable hypha of the transgenic isolate Rcc-8B9-GFP carrying rubellin vesicles

Ramularia leaf spot has emerged as a newly important disease of barley associated with significant grain yield and quality losses across Europe and a number of other temperate regions of the world [1]. The disease was first recognised in 1893 in Italy and the fungal pathogen first described as *Ophiocladium hordei* [2]. Sutton and Waller [3] reclassified this ascomycete fungus to the genus *Ramularia*, within the family Mycosphaerellaceae in the class Dothideomycetes and proposed the species name *R. collo-cygni* because of the distinctive swan’s neck-like shape of the fungal conidiophores (Fig. 1a, b). This systematic position was subsequently supported by phylogenetic analysis of fungal ribosomal DNA sequences [4, 5].

The importance of Ramularia leaf spot as a disease of barley has become more apparent in recent years as reliable methods for isolation and detection of *R. collo*-*cygni* have become available [6–10]. Ramularia leaf spot is often confused with physiological leaf spotting and leaf spotting diseases, such as net blotch caused by the fungus *Pyrenophora teres*, but is distinguished by characteristic reddish-brown, rectangular lesions visible on both sides of the leaf and surrounded by a chlorotic halo (Fig. 1c). Recently reclassified as a major pathogen of its main host plant barley (*Hordeum vulgare*), *R. collo-cygni* has also been reported to colonise wheat, rye, oats, maize and many other grass species including the model *Brachypodium distachyon* [11, 12]. *R. collo-cygni* is transmitted both via spores and through infected seeds [6, 13], and exhibits intercellular, apoplastic colonisation of its host [10, 14, 15]. Disease is typically observed late in the growing season after the crop flowers, suggesting a link between Ramularia leaf spot symptom expression and host developmental stage [16]. However, *R. collo-cygni* can also colonise the host asymptomatically (Fig. 1d; [13]). Expression of Ramularia leaf spot symptoms has been linked to host genetics [17–19] but, as with many other Dothideomycete diseases, expression of symptoms has a strong environmental component, in particular responding to light intensity [8, 20–22]. This lifestyle, asymptomatic vertical transmission through seeds combined with the ability to switch lifestyle when faced with an adverse host environment, is reminiscent of that followed by many plant endophytes [23, 24]. Indeed, it was proposed that *R. collo-cygni* was ancestrally an endophyte and that pathogenicity is a more recent evolutionary phenomenon [25]. Whether changes in pathogen or host genetics or agronomic practices have resulted in the emergence of Ramularia leaf spot as an important pathogen of barley since the late 1990s is currently unclear.

Many Dothideomycetes produce secondary metabolites that are involved in fungal pathogenesis and/or virulence [26]. *R. collo*-*cygni* produces a number of anthraquinone-derived secondary metabolites called rubellins [27, 28]. Rubellin D, the most widely studied of these compounds, is a light activated, nonhost specific phytotoxin [26]. Rubellins are likely to be synthesised through a polyketide synthase pathway [29], similar to toxin production in other Dothideomycetes [26, 30, 31]. Rubellin D may act by increasing reactive oxygen species (ROS) production resulting in α-linolenic acid peroxidation, leaf chlorosis and necrosis [27, 28]. How *R. collo*-*cygni* produces these toxic secondary metabolites and what roles rubellins have in fungal colonisation and disease expression remains to be determined.

To address these and other questions relating to the biology of this fungus and its phylogenetic position within the Dothideomycetes, we have sequenced and assembled the genome of *R. collo*-*cygni* isolate DK05 Rcc001 (isolated from a susceptible host). We compare the genome to those of other plant pathogens including *Zymoseptoria tritici* [syn. *Mycosphaerella graminicola*], *Pseudocercospora fijiensis* [syn. *Mycosphaerella fijiensis*] and *Dothistroma septosporum* [syn. *Mycosphaerella pini*] from the Mycosphaerellaceae. The high-quality genome of *R. collo*-*cygni* provides a foundation for future studies aimed at understanding why Ramularia leaf spot has emerged as an important threat to barley production.

## Results and discussion

### Genome assembly and annotation of gene models

The 30.3 Mb *R. collo*-*cygni* genome was sequenced using a combination of Illumina and Roche 454 FLX technologies to 90-fold coverage and assembled into 576 contigs ranging from 200 bp to 1,386,477 bp in size (Table 1). There were 355 contigs greater than 1 kb in length which had an average GC content of 51.5 % and accounted for 30.1 Mb of the assembly (N50 = 201,222 bp). We predicted 11,617 protein-coding gene models, of which 8514 had transcript evidence from RNAseq analysis of in vitro fungal cultures (see Methods). The average coding sequence length was 1423 bp, with a maximum length of 21,156 bp. Both the estimated *R. collo*-*cygni* genome size and number of predicted gene models are similar to those of other Dothideomycetes [32] including *Z. tritici* [33] and *D. septosporum* [30]. The genome appears relatively complete, with 94 % of the 248 core eukaryotic gene models in the CEGMA toolkit judged to be complete in the assembly. A genome browser presenting the *R. collo*-*cygni* genome data can be found at http://ramularia.org/jbrowse and the sequence data has been submitted to the European Nucleotide Archive [http://www.ebi.ac.uk/ena/data/view/PRJEB11432].

**Table 1:** General features of the *Ramularia collo*-*cygni* isolate DK05 Rcc001 genome assembly

Genome size (bp)	30,300,614
Coverage	90x
Average GC (%)	51.4
Total number of contigs	576
Number of contigs >1 kb	355
N50 (bp) contigs >1 kb	210,222
Max contig size (bp)	1,386,477
Min contig size (bp)	200
Total number of coding sequences (CDS)	11,617
Average length of CDS (bp)	1423
Average coding density^a^	0.546077

^a^Average coding density = Total number of CDS bases/Total genome bases

**Fig. 2:**
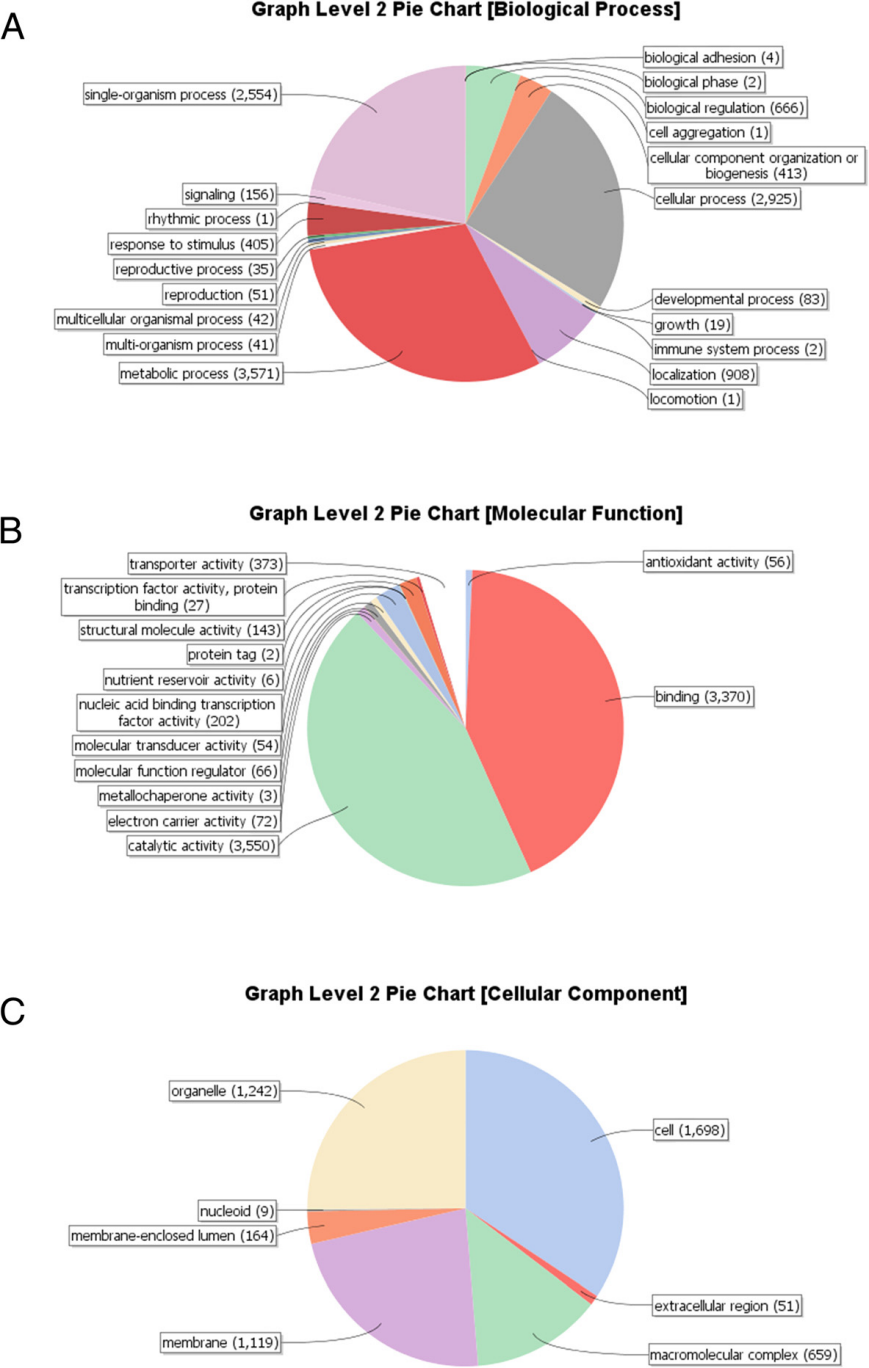
Distribution of Blast2GO gene ontology (GO) categories from the putative *Ramularia collo*-*cygni* gene model annotation. **a** Biological processes, **b** Molecular function, **c** Cellular component

Each predicted gene model was annotated using Blast2GO (Additional file 1) [34, 35]. Most of the top BLASTp matches for the predicted protein set were to *Z. tritici* (36.5 %), *D. septosporum* (15.8 %) and *P. fijiensis* (11.2 %), as expected from their phylogenetic relatedness (Additional file 2). One sixth (1989 gene models; 17.1 %) had no significant matches in the NCBI nr database. For 7442 *R. collo*-*cygni* gene models we gathered 24,526 level 2 gene ontology (GO) terms which were classified into the categories biological process, molecular function and cellular component (Fig. 2).

### Phylogenetic relationships of *R. collo*-*cygni* and Dothideomycete fungi

**Fig. 3:**
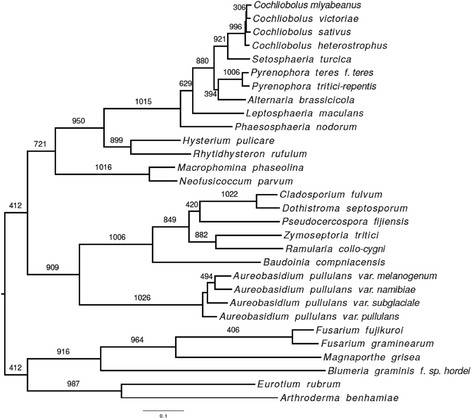
Phylogenetic relationships between *Ramularia collo-cygni* and 29 other fungi with sequenced genomes. Maximum likelihood phylogenetic tree based on a supermatrix analysis of 1026 proteins from 30 taxa (24 Dothideomycetes and six other ascomycetes: see Methods). Branch support is shown as the number of gene trees out of 1026 that supported the presented topology. Bootstrap values are 99–100 for each branch and therefore not shown. Branch length corresponds to a mean estimate of substitutions per site as indicated by the scale bar

We identified 1026 orthologous protein sets that were largely single-copy across 30 fungal taxa, focusing on Dothideomycetes. Phylogenetic analysis of these alignments clearly positioned *R. collo*-*cygni* within the order Capnodiales in the Mycosphaerellaceae (Fig. 3). Within the Mycosphaerellaceae *R. collo*-*cygni* was sister to *Z. tritici*. The other three Mycosphaerellaceae assessed (*P. fijiensis*, *C. fulvum* and *D. septosporum*) formed a sister clade to *R. collo*-*cygni* plus *Z. tritici,* congruent with previous, less-complete studies which did not include *R. collo*-*cygni* [30, 32].

### The *R. collo*-*cygni* secretome and candidate effector proteins

We identified 1053 genes encoding putative secreted proteins, approximately 9 % of the *R. collo*-*cygni* proteome, ranging from 45 to 2512 amino acids in length. The predicted *R. collo*-*cygni* secretome is similar in size to secretomes of other Dothideomycetes such as *Z. tritici* [33, 36] and *D. septosporum* [30]. Most of the predicted secreted proteins (854; 81 %) had significant sequence similarity (BLASTp E-values less than 1e^−6^) to proteins from other organisms, with matches to *Z. tritici* (324; 30.7 %), *P. fijiensis* (127; 12 %), *D. septosporum* (122; 11.6 %) and *Sphaerulina musiva* (94; 8.9 %) most common. Most (59.6 %) of the predicted secreted proteins were annotated with GO terms, the commonest being “oxidation and reduction”, “carbohydrate metabolism” and “proteolysis” in the biological process hierarchy, and “hydrolase activity including hydrolysis of carbohydrates” and “proteins with redox related functions including oxidoreductase and peroxidase activity” in the molecular function hierarchy (Additional file 3). In the *Z. tritici* secretome the protein family domain PF01238, corresponding to peroxidase_2 family or chloroperoxidase, was abundant [36]. Transcriptome profiling during *Z. tritici* infection highlighted up-regulation of chloroperoxidase genes during asymptomatic intercellular growth of the fungus [37]. This family was also found to be expanded in genomes of other plant pathogens within the Mycosphaerellaceae compared to other plant pathogenic fungi [36]. Matches to this domain were found in 21 different proteins in the *R. collo*-*cygni* secretome (Additional file 3). Most other Mycosphaerellaceae fungi have 15 or more chloroperoxidase genes whereas most other plant pathogenic fungi including Dothideomycetes of the order Pleosporales typically have less than 10 of these genes [36]. This expanded family of chloroperoxidases may play an important role during the endophytic stage of *R. collo*-*cygni* colonisation.

Many experimentally validated plant pathogen effectors are secreted, cysteine-rich, low molecular weight proteins, termed small secreted proteins (SSP; [38]). A total of 150 proteins from the predicted *R. collo*-*cygni* secretome matched these criteria (Additional file 4). Effectors are frequently pathogen-specific and just over half (78) of the *R. collo*-*cygni* SSPs had no significant similarity to proteins found in public databases, suggesting they are unique to this species. Previously reported estimates of the proportion of species-specific SSPs across Dothideomycetes range from 20 to 30 % [32, 39]. The rest of the *R. collo*-*cygni* SSPs (48 %) had significant similarities to proteins from other organisms, in particular *Z. tritici* (24; 33.3 %), *S. musiva* (12; 16.7 %) and *P. fijiensis* (10; 13.9 %). Only 18 (12 %) SSPs had predicted PFAM domains, reflecting the typically unknown function of effector proteins. Sixty-seven (45 %) of the predicted SSPs had transcript support from in vitro grown fungal mycelial RNASeq. *R. collo*-*cygni* SSP gene models that had no transcript support are of particular interest as they may only be expressed *in planta*. Overall, while there appears to be a degree of sequence conservation between secreted proteins of *R. collo*-*cygni* and other *Mycosphaerella* fungi the function of these putative effector proteins in the biology of the different diseases is currently unknown. Variation between repertoires of putative effector proteins of each species are likely to contribute to the distinct biology of these pathogens. It should also be noted that the analysis of the putative effector content of the *R. collo*-*cygni* genome may not have been exhaustive. Evidence from the genome of the obligate biotroph *B. graminis* f. sp. *tritici* has indicated the presence of putative effector proteins that do not contain a signal peptide suggesting that they are either non-secreted or secreted through an alternative pathway [40]. Furthermore, plant pathogen effectors are often associated with genomic regions rich in repetitive DNA [41]. Due to the small nature of effector proteins it is possible that the gene annotation process may not have detected them. As such further analysis of the putative effector complement and subsequent characterisation of species-specific SSPs that are specifically expressed *in planta* may provide further clues to the mechanisms of *R. collo*-*cygni* pathogenicity.

### Pathogenicity and virulence genes

Fungi use diverse infection strategies for host colonisation and fungal development. We used the Pathogen-Host Interaction database, PHI-base [42, 43], to determine the presence in *R. collo*-*cygni* of orthologues of pathogenicity genes experimentally confirmed in other fungal species. A total of 1291 *R. collo*-*cygni* gene models matched 547 PHI-base accessions (Additional file 5). These genes encoded transporters, transcription factors, secondary metabolite biosynthesis enzymes and previously characterised effectors from other pathogens, including three BEC-type effectors from *B. graminis* f. sp. *hordei* [44]. Of particular interest was the identification of 73 *R. collo*-*cygni* gene models that matched known Dothideomycetes pathogenicity determinants that are associated with toxin production. We identified putative orthologues of proteins involved in biosynthesis of the host-specific HC-toxin (PHI:97; PHI:157) and T- toxin (PHI:2834; PHI:2835; PHI:2836; PHI:2837; PHI:2838; PHI:2839) from *Cochliobolus* spp. and the AK-toxin (PHI:133; PHI:134; PHI:2831; PHI:2832), ACR-toxin (PHI:2608) and ACT-toxin (PHI:2431) produced by *Alternaria alternata* [26] were identified in the *R. collo*-*cygni* genome. Putative orthologues of genes involved in the biosynthesis (PHI:1046; PHI:1047; PHI:1048; PHI:1049; PHI:1050; PHI:1051) and transport (PHI:141) of the cercosporin toxin produced by *Cercospora* spp. [45] were also identified. Cercosporin is a perylenequinone compound that is a light activated nonhost specific pathogenesis-related toxin [46]. The rubellin toxins produced by *R. collo*-*cygni* also cause light-activated *in planta* necrosis, fatty acid peroxidation and ROS production [28]. Although to date the only toxins characterised in *R. collo*-*cygni* are the rubellins, it is probable that this fungus produces a complex arsenal of toxins to facilitate host colonisation and niche exploitation. Determining whether *R. collo*-*cygni* produces toxins related to cercosporin or toxins characterised in other Dothideomycetes is an important research goal.

### Carbohydrate-active enzymes

**Fig. 4:**
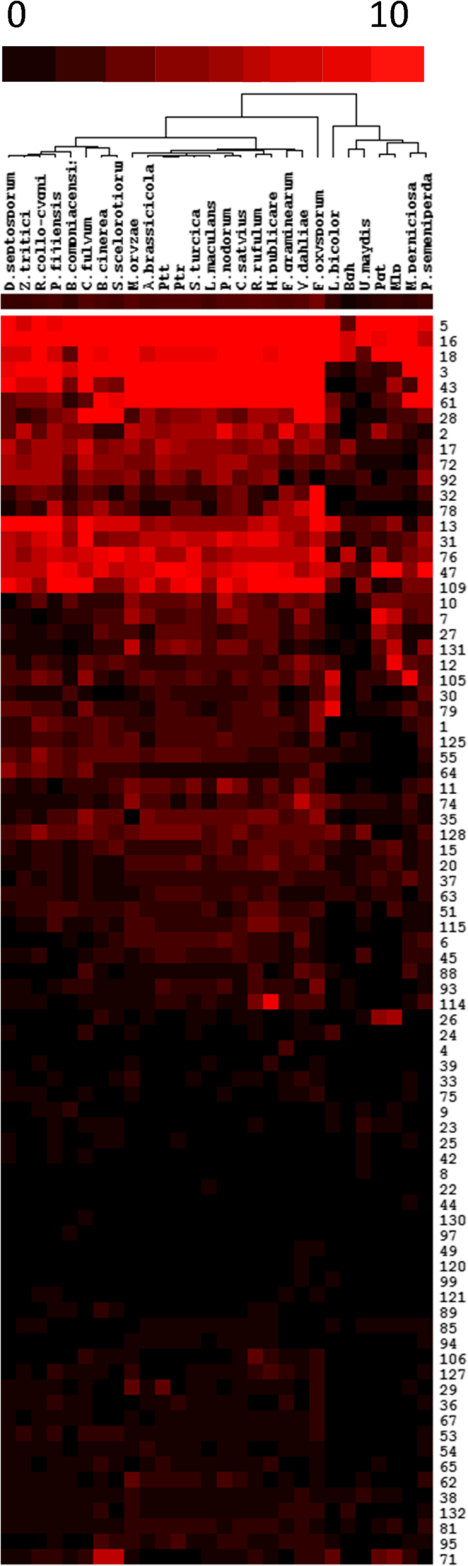
Comparison of *Ramularia collo*-*cygni* glycoside hydrolase (GH) family complement with 27 other fungal species. GH families and fungal species are hierarchically organised with the heat map indicating the number of members in each family (*Black* = 0, *bright red* = >10). Ptt = *Pyrenophora teres* f. sp *teres*; Ptr = *Pyrenophora tritici-repentis*; Bgh = *Blumeria graminis* f. sp *hordei*; Pgt = *Puccinia graminis* f. sp *tritici*; Mlp = *Melampsora larici-populina*

**Fig. 5:**
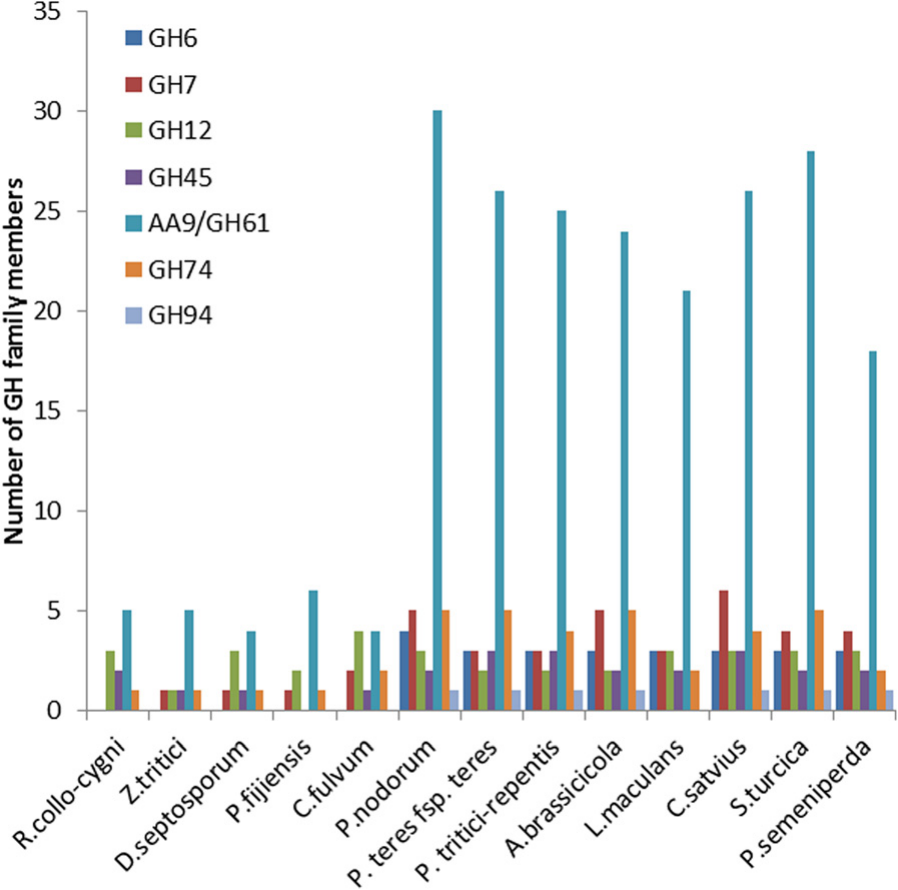
Complement of *Ramularia collo*-*cygni* cellulose degrading enzymes compared to other Dothideomycete fungi

Fungal pathogens need to derive carbohydrates from their hosts and therefore express an array of enzymes capable of metabolising different carbohydrate substrates. The diversity of carbohydrate-active enzymes (CAZymes) can provide insights into the biology of fungal interactions with their specific hosts [47]. *R. collo*-*cygni* had a total of 520 CAZymes, 226 of which were predicted to be secreted. We identified 223 glycoside hydrolases (GH), 5 polysaccharide lyases (PL), 101 carbohydrate esterases (CE), 55 auxiliary activities (AA), 107 glycosyltransferases (GT) and 29 carbohydrate-binding modules (CBM; Additional file 6). The overall CAZyme complement of *R. collo*-*cygni* is consistent with that observed in other Dothideomycetes [32, 47]. GH enzymes hydrolyze bonds linking carbohydrates to other molecules [48]. Specific GH family members act on different polysaccharide components of plant cell walls such that the genomic complement of these enzymes can be associated with the trophic habit of fungal pathogens [33, 47]. The GH family complement was compared to that of 27 other fungal species with differing trophic strategies including other Dothideomycetes, Ascomycetes and Basidiomycetes [32]. Cluster analysis of the GH family positioned the Capnodiales in a cluster distinct from the Pleosporales (Fig. 4) in agreement with previous reports [32, 47]. Within the Capnodiales cluster *R. collo*-*cygni* was placed in a sub-cluster together with the *Mycosphaerella* species (Fig. 4). Further inspection of the GH complement of *R. collo*-*cygni* highlighted that *Mycosphaerella* species and *R. collo*-*cygni* have a reduced complement of cellulose-degrading GH enzymes compared to other Dothideomycetes (Figs. 4 and 5). In particular, *R. collo*-*cygni* has a severely reduced complement of AA9 (formerly known as GH61) copper-dependent, lytic polysaccharide monooxygenase enzymes and no GH6 (endoglucanse and cellobiohydrolase) or GH7 (β-1,4-glucanase, endo-β-1,3-1,4-glucanase, reducing end-acting cellobiohydrolase, and chitosanase) enzymes. The GH7 family members were present in all of the other fungi examined except the biotrophs *B. graminis* f. sp *hordei* and *Ustilago maydis*, and the symbiont *L. bicolor* (Fig. 4; Additional file 6).

Many economically important fungal pathogens of the genus *Mycosphaerella* have long latent periods of asymptomatic growth before disease occurs [20, 49–51]. As such these fungi need to avoid detection by the host’s defence system to allow successful colonisation. *Z. tritici* and *C. fulvum* secrete carbohydrate-binding module 50 (CBM50) domain proteins containing LysM motifs that act to sequester chitin and interfere with chitin-triggered host immunity [52, 53], preventing recognition of the pathogen by the host chitin receptors CERK1 and CEBiP1 [54]. *R. collo*-*cygni* has at least three CBM50 containing proteins (Table S5) which is in the range for most Dothideomycetes except for *Colletotrichum* spp. where gene expansion of the CBM50 family has been reported [55, 56].

Plant immune systems can recognise and degrade fungal chitin, and some species display active remodelling of the cell wall during invasion. CBM14 and CBM18 family proteins bind chitin [57], and the CBM14-domain effector protein AVR4 from the biotroph fungus *C. fulvum* can bind chitin at the fungal cell wall to prevent its enzymatic degradation by chitinases [58]. No CBM14 family proteins were detected in the *R. collo*-*cygni* genome. A lack of AVR4-like proteins was also reported for *Z. tritici* where instead the CBM50 containing Mg1LysM and Mg3LysM proteins are able to bind chitin and protect fungi from hydrolytic chitinases [52]. *R. collo*-*cygni* has six CBM18 family proteins, similar to other *Mycosphaerella* species, but compared to other Dothideomycetes, *R. collo*-*cygni* and the Capnodiales typically have a contracted CBM18 domain family. Some biotrophic rust fungi and *Colletotrichum graminicola* have been shown to convert chitin in the fungal cell walls of hyphae growing on the leaf surface to chitosan, through the action of chitin deacetylase (CE4) and chitin synthase (GT2), once invasive hyphae penetrate the leaf surface [59], and this may prevent host chitinases from digesting invading hyphae. *R. collo*-*cygni* has twenty-one GT2 proteins which is at the higher end of the range observed in Dothideomycetes but similar to the GT2 complement of *P. fijiensis* and the extremophilic saprotroph *Baudoinia compniacensis* [60]. *R. collo*-*cygni* also has six CE4 proteins, the same number found in *Z. tritici*, *D. septosporum*, *P. fijiensis* and *C. fulvum*, but fewer than in any of the Pleosporales fungi (Additional file 6).

The reduced number of plant cell wall degrading enzymes in *R. collo*-*cygni* coupled with the presence of genes with known roles in avoiding chitin recognition by host defence systems suggests that this species may also utilise “stealth pathogenesis”, as proposed for *Z. tritici* [33]. *R. collo*-*cygni* is frequently detected in the field in the absence of visible disease [6]. Fungal biomass can build up during asymptomatic development suggesting the fungus is able to avoid recognition by the host [19]. Expression of Ramularia leaf spot symptoms in hosts has a host genetic component [17, 18, 61] but is also modulated by changes in host abiotic stress [8, 12] and development [16]. These features of the pathogenesis of Ramularia leaf spot, combined with the vertical transmission of *R. collo*-*cygni*, suggest that this species is actually an endophyte that only becomes pathogenic under specific conditions [1, 25]. The closely-related *Z. tritici* is proposed to have evolved from an endophytic ancestor [33], and *R. collo*-*cygni* may be an endophyte in which pathogenicity has evolved more recently [25]. Determining the roles that CAZymes play during the symptomless and necrotrophic phases will provide valuable insights into the fungal and host cues that trigger *R. collo*-*cygni* switching from endophytic to pathogenic development.

### Peptidases

Peptidases have multiple functions in plant pathogens including degradation of host defence proteins, signalling and nutrition. *R. collo*-*cygni* was predicted to have 365 putative peptidases of which 46 % were serine peptidases. Metallopeptidases (87; 23.8 %) and cysteine peptidases (64; 17.5 %) were also highly represented, whereas lower numbers of aspartic (18; 4.9 %) and threonine (20; 5.5 %) peptidases and single glutamic and N9 asparagine peptidases were identified. Five gene models were classified as peptidase inhibitors (Table 2). The distribution of peptidase classes observed in *R. collo*-*cygni* was similar to that in *Z. tritici*, *P. fijiensis* and *D. septosporum* (Table 2). Ninety-four (25.8 %) peptidases were predicted to be secreted, and most of these were either serine (55; 32.5 %) or metallo-peptidases (21; 24.1 %). Typically serine, metallo- and cysteine peptidases are the most prevalent types in Dothideomycetes whereas asparagine peptidases are relatively uncommon [32, 39]*.*

**Table 2:** Peptidase complement of *Ramularia collo*-*cygni*, *Zymoseptoria tritici*, *Dothistroma septosporum* and *Pseudocercospora fijiensis*

	Aspartic	Cysteine	Glutamic	Inhibitor	Metallo	Asparagine	Serine	Threonine	Total
*R. collo-cygni*	18	64	1	5	87	1	169	20	365
*Z. tritici*	22	53	4	7	79	0	155	18	338
*D. septosporum*	15	49	1	6	75	0	160	19	325
*P. fijiensis*	15	65	2	6	84	0	173	21	366

Data based on [32]

### Secondary metabolites and rubellin toxin biosynthesis cluster

Many plant pathogenic fungi produce a wide range of secondary metabolites, some of which have important roles in virulence and disease. These are derived from four core biosynthetic origins: polyketide synthases (PKS); non-ribosomal peptide synthases (NRPS); terpene cyclases (TC; syn. terpene synthase) and dimethylallyl tryptophan synthases (DMATS) [26]. *R. collo*-*cygni* produces nonhost-specific photodynamic anthraquinone toxins, called rubellins [27–29] that are most likely derived from polyketides [29]. Within the *R. collo*-*cygni* genome 19 PKS, fourteen NRPS and four TC were located (Table 3). No DMATS were identified. *R. collo*-*cygni* has similar numbers of NRPS and TC genes as other Dothideomycetes. However, the number of PKS in *R. collo*-*cygni* is nearly double that described for other members of the Capnodiales (Table 3; [30, 32]).

**Table 3:** Comparison of lifestyle and key secondary metabolism genes between *Ramularia collo*-*cygni* and other selected Dothideomycetes

	Lifestyle	Polyketide synthase (PKS)	Non-ribosomal peptide synthase (NRPS)	Terpene cyclase/synthase (TC/TS)
*Ramularia collo-cygni*	Endophyte/necrotroph	19	14	4
*Zymoseptoria tritici* ^a^	Hemibiotroph/necrotroph	11	9	5
*Dothistroma septosporum* ^a^	Hemibiotroph	6	7	7
*Pseudocercospora fijiensis* ^a^	Hemibiotroph	8	11	6
*Cladosporium fulvum* ^a^	Biotroph	10	12	5
*Stagonospora nodorum* ^a^	Necrotroph	19	10	7
*Pyrenophora teres* f.sp *teres* ^a^	Necrotroph	22	44	11
*Pyrenophora repentis-tritici* ^a^	Necrotroph	17	16	7

^a^Data based on [30] and [32]

**Fig. 6:**
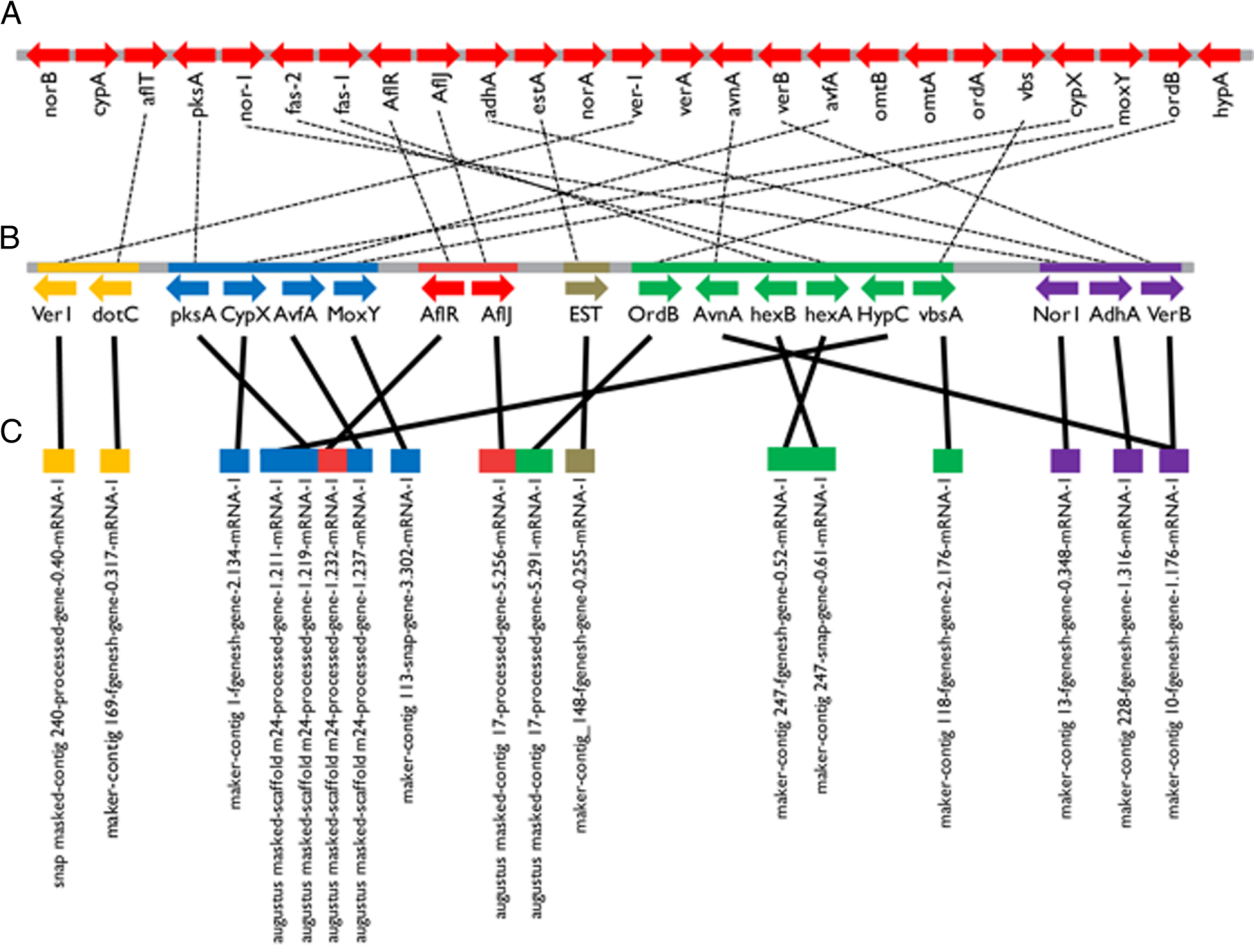
Arrangement of predicted toxin biosynthesis genes across *Aspergillus flavus*, *Dothistroma septosporum* and *Ramularia collo-cygni.* **a** *A. flavus* 70 kb Aflatoxin biosynthesis cluster [62, 63]. **b** *D. septosporum* dothistromin biosynthesis cluster fragmented across the 1.26 Mb chromosome 12 [30]. The six miniclusters are indicated by different colours: *Ver1* = orange; *PksA* = blue; *AflR*/*AflJ* = red; *EST* = brown; *VbsA* = green; *Nor1* = purple. Positons of clusters are not drawn to scale. **c** *R. collo-cygni* orthologs of *D. septosporum* dothistromin biosynthesis genes fragmented across multiple contigs/scaffolds

The rubellin toxins produced by *R. collo*-*cygni* are predicted to be synthesised through a polyketide-derived pathway [29]. Polyketide-derived toxins from other Dothideomycetes, such as the *D. septosporum*-produced dothistromin, are synthesised through pathways similar to that used to produce aflatoxin in *Aspergillus* spp. [22, 31, 62]. Biosynthesis of aflatoxin requires at least 25 enzymes as well as some regulatory proteins, and the genes encoding these are clustered together in a 70 kb region of the *Aspergillus* genome (Fig. 6; [63, 64]). An ancestral core cluster, required to form the initial polyketide product, has been proposed that comprises the genes *AfPksA*, *Affas-1*, *Affas-2* and *Afnor-1*, possibly along with regulatory genes *AfAflR* and *AfAflJ* [65]. Dothistromin has some structural similarity to the aflatoxin intermediate versicolorin B [31] and functional orthologues of aflatoxin biosynthesis genes have been described in *D. septosporum,* although the dothistromin metabolic cluster is fragmented into *DsPksA*, *DsVbsA*, *DsAflR*/*DsAflJ*, *DsEST*, and *DsVer1* mini-clusters, across a single chromosome (Fig. 6; [22, 30, 31, 66]). Homologs of most dothistromin biosynthesis genes have been found in other Dothideomycete genomes with the complete set identified in *C. fulvum*, a sister species to *D. septosporum*, even though no dothistromin production by this fungus has been reported [30, 67].

As *R. collo*-*cygni* produces the polyketide-derived rubellin toxins and due to the close phylogenetic relationship between *R. collo*-*cygni* and *D. septosporum* (Fig. 3) we used the protein sequences of known dothistromin and aflatoxin biosynthesis genes [30] to identify candidate toxin biosynthesis genes in *R. collo*-*cygni* (Additional file 7). Homologues of all 18 genes found in the six dothistromin biosynthetic mini-clusters [68] were identified (Additional file 7). However, reciprocal BLASTp analyses indicated that only nine of these proteins were likely to be orthologous to the canonical toxin production loci. The others were identified as orthologues of related enzymes, as has been reported from most other Dothideomycetes [32]. *D. septosporum DsAvnA* and *DsVerB* both had the closest similarity to the same *R. collo*-*cygni* gene model. The dothistromin biosynthetic clusters found in *D. septosporum* were not conserved in *R. collo*-*cygni* (Fig. 6). Two putative homologs of genes from each of the *DsPksA* and *DsVbsA* clusters appear to co-locate in the *R. collo*-*cygni* genome. *RccPksA* (a polyketide synthase) and *RccAvfA* (a NAD(P) reductase) were identified on the 229,962 bp scaffold m24 (Fig. 6), whereas *RccHexA* and *RccHexB*, fatty acid synthases from the *DsVbsA* cluster, were located on the 42,586 bp contig247 (Fig. 6). While the *R. collo*-*cygni* genome has not been assembled to chromosome level, the lack of observed synteny for these loci is telling and suggests that any toxins produced by *R. collo*-*cygni* are likely to be synthesized through pathways distinct from those in *D. septosporum*.

While *C. fulvum* contains the complete dothistromin biosynthetic pathway, the toxin is not known to be produced by this fungus, and essentially no expression of dothistromin-biosynthesis related transcripts has been observed *in planta* or in vitro [30]. We probed expression of the predicted *R. collo*-*cygni* homologs of dothistromin biosynthesis genes using RT-PCR analysis of in vitro and *in planta*. Expression was assessed in RNA extracted from 5, 10, 15 and 20 day old fungal cultures grown in Alkyl Ester broth (AEB) and from barley leaf samples collected from naturally infected field grown plants at two growth stages (GS78 and GS83). Ramularia leaf spot symptoms were clearly visible on plants at both growth stages with no significant difference (*P* = 0.440) in disease levels (Additional file 8) even though there was significantly less green leaf area retention at GS83 (*P* < 0.001; Additional file 8). All of the *R. collo*-*cygni* homologs of dothistromin biosynthesis genes, including the nine true orthologues and the closest *R. collo*-*cygni* gene models to remaining *D. septosporum* genes, were expressed in at least one of the in vitro time points (Additional file 7). *RccCypX*, *RccAvfA*, *RccMoxY*, *RccAflJ*, *RccOrdB*, *RccHypC* and *RccAdhA* transcripts were expressed *in planta* at GS78 but not at GS83 when green leaf area retention had declined, whereas *RccPksA*, *RccHexA* and *RccHexB* were expressed at both *in planta* growth stages (Additional file 9). *RccVer1*, *RccdotC*, *RccAflR*, *RccVbsA*, *RccNor1*, *RccAvnA*/*VerB* and *RccEST1* showed no *in planta* expression at the time points studied. It will be of interest to ascertain whether or not any of these genes are involved in the production of rubellin or other toxins or secondary metabolites that have important biological functions in *R. collo*-*cygni* development or the expression of disease symptoms.

**Fig. 7:**
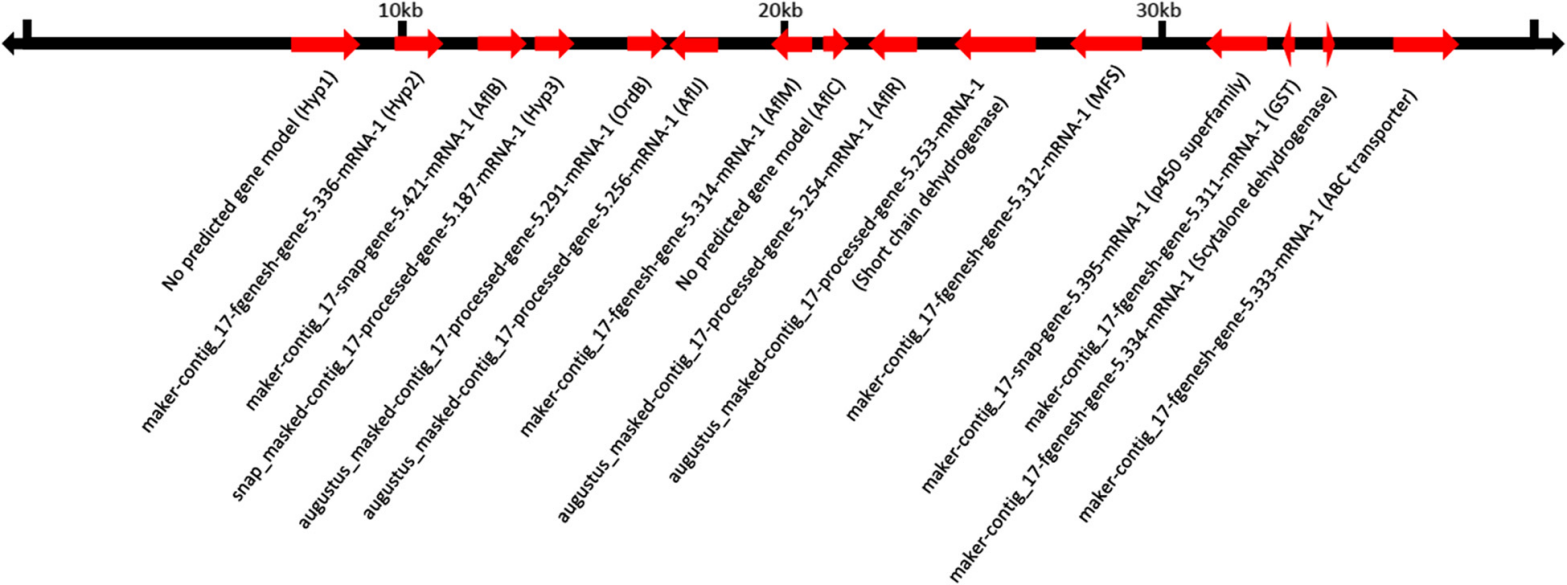
A predicted metabolic gene cluster in a 40 kb region on *Ramularia collo-cygni* Contig17. Putative gene functions based on BLASTp analysis are indicated below the gene models. Where no putative function could be assigned for a gene its function is designated as hypothetical (Hyp). Arrowheads indicate predicted direction of transcription for each open reading frame

*AfAflR* regulates transcription of most of the genes in the aflatoxin biosynthetic cluster [62] and the *D. septosporum DsAflR* orthologue regulates expression of genes involved in dothistromin biosynthesis [69] suggesting that AflR-like proteins may act as transcriptional regulators of toxin genes across fungal species. *AfAflR* is located in the middle of the aflatoxin biosynthesis gene cluster adjacent to the divergently transcribed *AfAflJ* which has also been shown to be involved in transcriptional regulation of aflatoxin biosynthesis [62]. In *Cercospora nicotianae*, the *AflR*-like *CTB8* and *AflJ*-like *CTB3* are components of the core cercosporin biosynthetic cluster [45]. *R. collo*-*cygni* homologs of the regulatory gene *DsAflJ* from the dothistromin *AlfR*/*AflJ* cluster and *DsOrdB*, an oxidoreductase, from the *DsVbsA* cluster were adjacent to each other on Contig17, the largest contig in the assembly (644 kb) containing a putative dothistromin biosynthesis gene homolog (Fig. 7). The 40 kb region surrounding *RccAflJ* and *RccOrdB* on Contig17 contained 13 gene models and two additional open reading frames (ORF), eight of which had significant similarity (BLASTp E-value less than 1e^−6^) to proteins from Dothideomycetes (Additional file 10). Four had matches to proteins with predicted functions including a putative ABC transporter, two short chain dehydrogenase/reductases and a scytalone dehydratase, all of which have been reported to have roles in the production of toxins, virulence and/or pathogenicity in Dothideomycetes and other fungi (Additional file 5; [70, 71]).

The remaining loci were most similar to hypothetical proteins from sequenced genomes, and had protein domain matches to short chain dehydrogenase/reductase, DUFF1772, major facilitator superfamily, glutathione S-transferase and p450 superfamily domains. Gene model augustus_masked-contig17-processed-gene-5.254-mRNA-1 was predicted to encode an *AflR*-like Zn2Cys6 transcription factor containing GAL4-like and AflR domains [62]. The biosynthetic roles of the genes on Contig17 remain to be determined, but all were expressed in barley leaves at GS78 showing Ramularia leaf spot symptoms whereas only the aldoketoreducatse-like *AflB1* and MFS-superfamily transporter genes were expressed in diseased tissues at GS83 (Additional file 9). Most of the Contig17 cluster genes were also expressed between 5–20 days during in vitro fungal growth except the p450, *AflC* and scytalone dehydratase-like genes (Additional file 9). Expression of many of the genes in this cluster in vitro and during disease symptom development prior to excessive leaf senescence may indicate a role for this cluster in fungal development before *R. collo*-*cygni* enters extensive necrotrophic growth. Functional analysis of the genes within the cluster on Contig17 will provide further insights into their biological role.

The high number of PKS genes in the genome suggests that *R. collo*-*cygni* may be able to produce other toxins in addition to the characterised rubellins [27–29]. An analysis of gene expression of various *R. collo*-*cygni* secondary metabolite biosynthesis genes under differing in vitro and *in planta* growth conditions will provide valuable insights into the roles these genes play in the biology of this organism. Experiments are underway to test whether the predicted orthologues of dothistromin/aflatoxin biosynthesis genes are involved in the production of secondary metabolites including rubellin in *R. collo*-*cygni*.

## Conclusions

Ramularia leaf spot recently emerged as an important disease of barley in temperate regions across the world. The 30.3 Mb genome of *R. collo*-*cygni* was predicted to contain 11,617 gene models, metrics consistent with the genome size and gene content of other Dothideomycetes. Phylogenetic analysis as well as observed similarities between *R. collo*-*cygni* and *Z. tritici*, *P. fijiensis* and *D. septosporum* at the gene level support classification of *R. collo*-*cygni* within the Mycosphaerellaceae [72]. Differences between the genomes of these fungi may hold the key to the unique pathology of *R. collo*-*cygni*. In particular identifying the fungal genes involved in the transition from endophytic growth to necrotrophy, the biosynthesis of the rubellin toxins and a further understanding of the genetic structure of *R. collo*-*cygni* populations [73] are important goals. Projects are currently underway within the *R. collo*-*cygni* research community to sequence additional genomes from geographically distinct isolates and those collected from non-barley hosts to improve our understanding of how this endophyte has recently established itself as the cause of a newly important foliar disease of barley.

## Methods

### Biological material

*R. collo*-*cygni* isolate DK05 Rcc001 was isolated in Denmark from the spring barley cv. Braemar, which is highly susceptible to Ramularia leaf spot [17], in 2005. Fungal cultures were maintained on potato dextrose agar (PDA, Sigma, Dorset, UK) plates at 16 °C as described previously [12]. Liquid cultures were prepared from an agar plug excised from a PDA plate with seven days fungal growth and placed in to 150 mL AEB [74] supplemented with 5 μg mL^−1^ streptomycin. Cultures were incubated at 16 °C under constant agitation at 120 rpm in the dark for 10–12 days. *R. collo*-*cygni* hyphae were filtered, freeze dried and ground to a fine powder prior to DNA extraction using the Illustra Nucleon PhytoPure Genomic DNA Extraction Kit (GE Healthcare Life Sciences, Little Chalfont, UK), according to the manufacturer’s instructions. Genomic DNA was resuspended in 1 % TE buffer (10 mM Tris, 1 mM EDTA- ethylenediaminetetraacetic acid) and subjected to phenol-chloroform-isoamyl alcohol purification. Contaminating RNA was removed by treating the DNA with RiboShredder ™ RNase blend (Cambio Ltd., Cambridge, UK) for 5 h at 37 °C followed by a further round of phenol-chloroform-isoamyl alcohol purification and resuspension of the DNA in 1 % TE. Total RNA was extracted from freeze dried DK05 Rcc001 hyphae scraped from 10 to 12 day old PDA culture plates grown as above using the Trizol (Life Technologies, Paisley, UK) method following the manufacturer’s instructions. Contaminating genomic DNA was removed using DNase-free kit as per the manual (Ambion, Leicestershire, UK).

### Genome assembly

The genome and in vitro transcriptome of *R. collo*-*cygni* were sequenced using a combination of Illumina Genome Analyser IIx and Roche 454 FLX Titanium technologies by the Edinburgh Genomics facility in the University of Edinburgh (https://genomics.ed.ac.uk/). Illumina 150 base sequencing generated 2.7 billion bases of genomic data from two libraries of 250 and 350 bp insert sizes and 1.3 billion bases of transcriptome data, and 454 sequencing 31.1 million genome and 3.4 million transcriptome bases (read lengths 100–500 bases). RNASeq data were assembled using the Trinity pathway (http://trinityrnaseq.github.io/) using default parameters. The COPE paired end connection tool [75] was used to merge the short insert Illumina reads and a draft assembly generated using the connected short insert library Illumina reads, the raw data from the longer insert library and the 454 data with CLC assembly cell (v.4.0.6 beta). This assembly was then scaffolded using the assembled transcriptome data using SCUBAT (B. Elsworth, G. Koutsopvoulos, unpublished). The assembled genome was assessed with REAPR ([76], where all fragment coverage distribution errors that scored above 0.5 were inspected by eye in Tablet [77]. The taxon annotated GC coverage plot approach (https://github.com/DRL/blobtools-light) was used to screen the assembly for contaminants (Additional file 11). Contigs with coverage below 9, one tenth of the average coverage, were removed from the assembled genome. Completeness of the draft genome assembly was assessed using CEGMA v.2.4 [78]. The MAKER (http://www.yandell-lab.org/software/maker.html) annotation pipeline was used to predict gene models. The final gene models are consensus sequences derived from MAKER using the gene prediction tools snap, genemark, augustus and fgenesh.

### Functional annotation

Protein sequences were compared using BLASTp (E-value threshold less than 1e^−6^) against the NCBI nr database and gene ontology (GO) terms assigned using the default settings in Blast2GO (v. 2.8.0; [34, 35]).

### Phylogenetic analysis

Proteomes of 25 Dothideomycetes including 18 plant pathogens and seven saprotrophs were downloaded from the Joint Genome Initiative (http://genome.jgi-psf.org/) for phylogenetic analysis. Six representative ascomycete proteomes (two Eurotiomycetes, one Leotiomycete and three Sordariomycetes) were also downloaded as outgroup species (Additional file 12; [30, 32, 33, 79–92]). OrthoMCL 2.0 [93] was used to identify gene clusters and 1737 single copy genes for the 31 downloaded fungal genomes plus *R. collo*-*cygni* were identified using the OrthoMCL output. Single copy genes were identified and aligned using Mafft linsi [94]. The alignments were masked using Zorro [95] with a cutoff value of 4.0 and checked for recombination using PhiPack [96] with default settings. After the recombination test 1251 alignments remained. Two taxa, *Cochliobolus carbonum* and *Cochliobolus heterostrophus* (C4), were removed prior to phylogenetic analyses due to high similarity with *Cochliobolus heterostrophus* (C5) and *Cochliobolus victoriae*, respectively. Two hundred and twenty five genes had several taxa with identical sequences and these were not analysed. Phylogenetic trees were inferred for each gene separately in RAxML [97, 98] using 20 starting trees and model estimation from alignment with PROTGAMMAAUTO. The resulting 1026 gene trees were summarised in a majority rule consensus tree calculated using Consense [99]. Rate of gene evolution was estimated from the branch lengths of the individual gene trees, and the genes were divided into an upper quartile of rapidly evolving genes, a lower quartile of slowly evolving genes, and one partition of medium rate genes. The three partitions were concatenated and analysed using RAxML with 100 rapid bootstraps and ML search from 20 starting trees with model PROTGAMMAAUTO for each partition. The individual gene trees were ultrametricised and analysed in DensiTree.

### Prediction of secretome and analysis of small secreted proteins (SSPs) and putative pathogenicity genes

The *R. collo*-*cygni* secretome was defined as the set of proteins with signal peptides but no transmembrane domain. Signal peptide and transmembrane domain predictions were derived from the InterProScan results of the Blast2GO analysis. SSPs were identified from the *R. collo*-*cygni* secretome as proteins smaller than 200 amino acids with >2 % cysteine residues. *R. collo*-*cygni* homologues of experimentally validated pathogenicity genes were identified using PHI-base v. 3.6 (http://www.phi-base.org/; [42, 43]). The database was searched using the *R. collo*-*cygni* gene models using BLASTp with an E-value threshold value of 1e^−10^. Only PHI-base entries characterised as being associated with reduced virulence, hypervirulence, loss of pathogenicity, a mixed outcome or predicted effectors were included in the analysis.

### Carbohydrate active enzymes (CAZymes) annotation

*R. collo*-*cygni* genes encoding CAZymes were identified using the dbCAN database (http://csbl.bmb.uga.edu/dbCAN/index.php) with default settings. The GH content of *R. collo*-*cygni* was compared with the GH family complement of 27 other fungal species (Table S9; [30, 32, 33, 79, 80, 83–85, 89, 90, 92, 100–107]) using hierarchical clustering. Fungal GH families were clustered using Cluster 3 [108] with a Euclidean distance matrix and complete-linkage clustering. Data were visualized with Treeview v.1.0.13 (http://www.eisenlab.org/eisen/?page_id=42).

### Peptidases

*R. collo*-*cygni* peptidases were predicted via BLASTp queries of the MEROPS database (http://merops.sanger.ac.uk/index.shtml) using default settings. For comparative purposes the peptidase complements of *Z. tritici*, *D. septosporum* and *P. fijiensis* were also predicted. Proteins were considered peptidases using a threshold E-value of 1e^−5^ [109]. Where multiple peptidases could be assigned to a gene model, the hit with the most significant E-value was selected. *R. collo*-*cygni* secreted peptidases were identified by cross referencing gene models with the InterProScan scan results from Blast2GO.

### Secondary metabolite annotation and toxin biosynthesis cluster analysis

PKS, NRPS, TC and DMATS genes were identified using BLASTp searches of protein models against the NCBI nr protein database (http://blast.ncbi.nlm.nih.gov/Blast.cgi). Genes putatively involved in the biosynthesis of toxins were identified using BLASTx and BLASTp searches of the *R. collo*-*cygni* genome sequence and protein models, respectively, using candidate sequences known to be involved in the biosynthesis of aflatoxin [62, 63] and dothistromin [30]. Contig17 was analysed as a potential gene cluster for additional genes involved in toxin production using the StarORF application (HTML version; Massachusetts Institute of Technology, USA). Putative open reading frames (ORF) were identified on Contig17 and the protein sequences screened against the NCBI nr protein database. Each putative ORF was confirmed by BLASTp analysis back against the *R. collo*-*cygni* gene models.

Gene specific primers were designed for candidate toxin biosynthesis genes using Primer3 (http://primer3.ut.ee/). Transcript expression was assessed during in vitro and *in planta R. collo*-*cygni* growth. In vitro expression was measured in fungal hyphae grown in AEB cultures harvested at 5, 10, 15, 20 days and *in planta* expression was measured in Ramularia leaf spot infected spring barley flag -1 (F-1) leaves collected from a field trial experiment (see Field experiment sampling). Total RNA was extracted as described above and cDNA was synthesised from 1 μg of total RNA using the Superscript III system (Invitrogen, Carlsbad, CA, USA) and target expression assessed using end point RT-PCR. cDNA was diluted 20-fold in dH_2_O and 5 μL used to amplify each target using gene specific primers (Additional file 13) and the Hotstar Master mix. Target sequences were amplified using a TProfessional Standard Thermocycler (Biometra, Göttingen, Germany) under the following cycling conditions: 15 min enzyme hot start activation at 95 °C followed by 35 cycles of 1 min denaturation at 94 °C, 1 min primer annealing at 60 °C and 1 min extension at 72 °C and a final extension cycle of 10 min at 72 °C.

### Field experiment sampling

Leaves of the spring barley cv. Concerto exhibiting Ramularia leaf spot symptoms were collected from a 10 m × 2 m plot of a field trial sown at the Bush Estate, Midlothian, Scotland in 2014. Herbicide and fertiliser treatments in the trial followed local practice with the sampled plot treated with Prothioconazole (0.44 L ha^−1^, Proline 275) at GS21-35 and GS41. F-1 leaf samples were collected at growth stages GS78 and GS83 and Ramularia leaf spot infection and green leaf area retention of each sample recorded as a percentage of leaf area covered. Leaf samples were stored at −80 °C for fungal gene expression analysis as described above (see Secondary metabolite annotation and toxin biosynthesis cluster analysis section).

## Abbreviations

AA, auxiliary activities; AEB, alkyl ester broth; CAZymes, carbohydrate-active enzymes; CBM, carbohydrate-binding modules; CE, carbohydrate esterases; DMATS, dimethylallyl tryptophan synthases; DUF, domain of unknown function; GH, glycoside hydrolase; GO, gene ontology; GS, growth stage; GT, glycosyltransferases; NRPS, non-ribosomal peptide synthases; ORF, open reading frame; PDA, potato dextrose agar; PKS, polyketide synthases; PL, polysaccharide lyases; ROS, reactive oxygen species; SSP, small secreted protein; TC, terpene cyclases

## Additional files

Additional file 1: Table S1. Blast2GO annotation of *Ramularia collo*-*cygni* gene models. (XLSX 1285 kb) Additional file 2: Figure S1.
*Ramularia collo*-*cygni* gene model top species hits from BLASTp analysis. (TIF 1644 kb) Additional file 3: Table S2. Predicted secretome of *Ramularia collo-cygni*. (XLSX 152 kb) Additional file 4: Table S3. Small secreted protein complement of *Ramularia collo-cygni* genome. (XLSX 43 kb) Additional file 5: Table S4. Putative *Ramularia collo*-*cygni* pathogenicity and virulence genes identified by analysis of PHI-base. (XLSX 96 kb) Additional file 6: Table S5. Carbohydrate active enzyme (CAZyme) complement of *Ramularia collo*-*cygni* genome compared to selected other fungi. (XLSX 43 kb) Additional file 7: Table S6. Homologs of dothistromin and aflatoxin biosynthesis genes in *Ramularia collo*-*cygni* genome. (XLSX 12 kb) Additional file 8: Figure S2. Data from naturally infected Ramularia leaf spot spring barley field trials. A. Ramularia leaf spot levels on cv. Concerto at GS78 and GS83 (% of the total leaf area). B. Green leaf area retention of Ramularia leaf spot infected samples at GS78 and BS83. (TIF 902 kb) Additional file 9: Table S7. RT-PCR analysis of *Ramularia collo*-*cygni* homologs of dothistromin biosynthesis genes and predicted secondary metabolism genes on Contig17. (XLSX 13 kb) Additional file 10: Table S8. BLASTp top hits for putative metabolic cluster genes on Contig17. (XLSX 11 kb) Additional file 11: Figure S3. Taxon annotated GC coverage plot of *Ramularia collo*-*cygni* genome assembly. (TIF 1542 kb) Additional file 12: Table S9. Details of fungal genomes used for comparative genomics in this study. (XLSX 17 kb) Additional file 13: Table S10. RT-PCR primer details. (XLSX 13 kb)
