# Supplementary figures and images for: The genome of the emerging barley pathogen Ramularia collo-cygni
Source: BMC Genomics. 2016 Aug 9;17:584. doi: 10.1186/s12864-016-2928-3 (PMC4979122; doi:10.1186/s12864-016-2928-3)

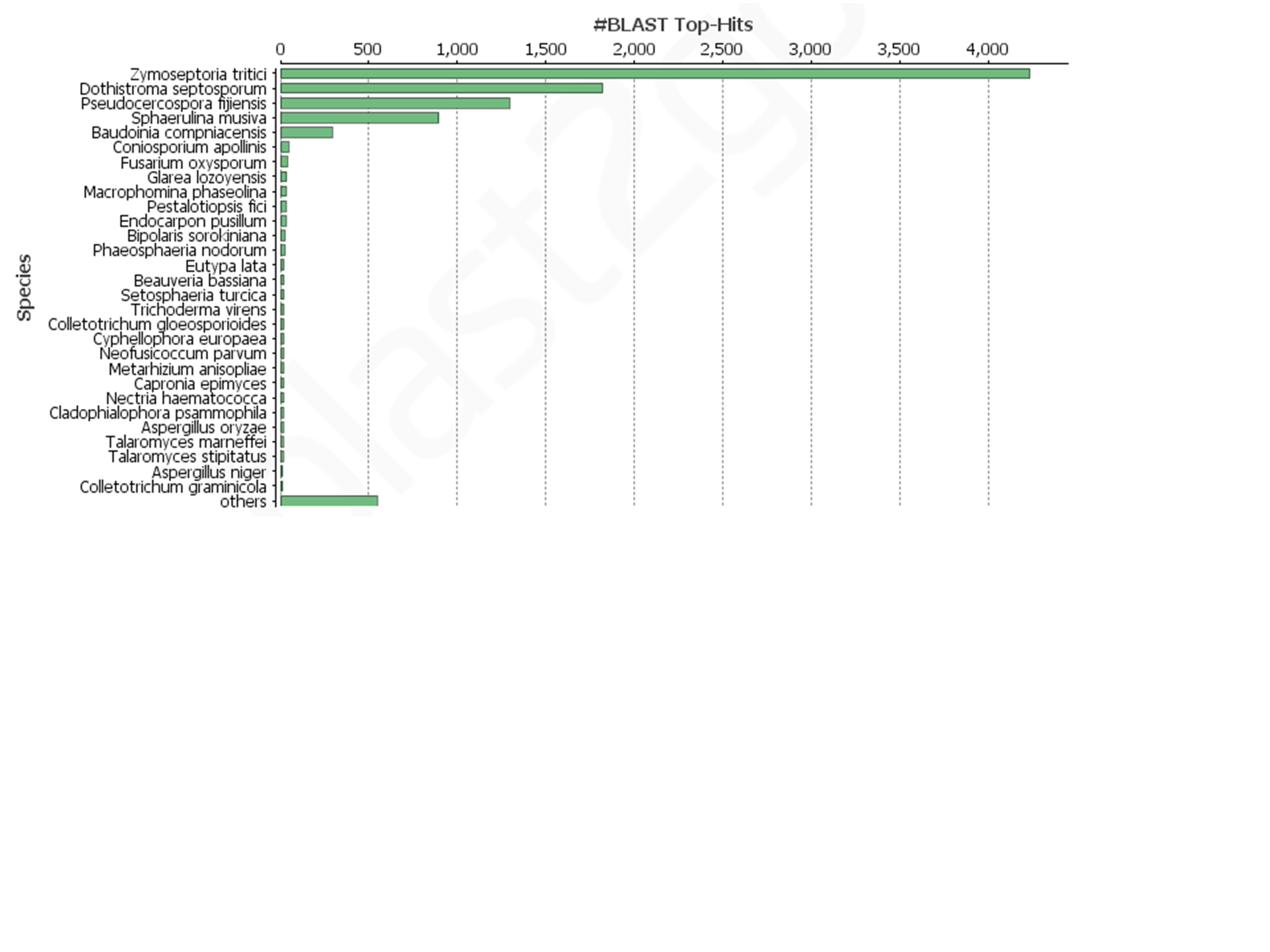

Supplement: Additional file 2: Figure S1. — Ramularia collo-cygni gene model top species hits from BLASTp analysis. (TIF 1644 kb) [file 12864_2016_2928_MOESM2_ESM.tif]

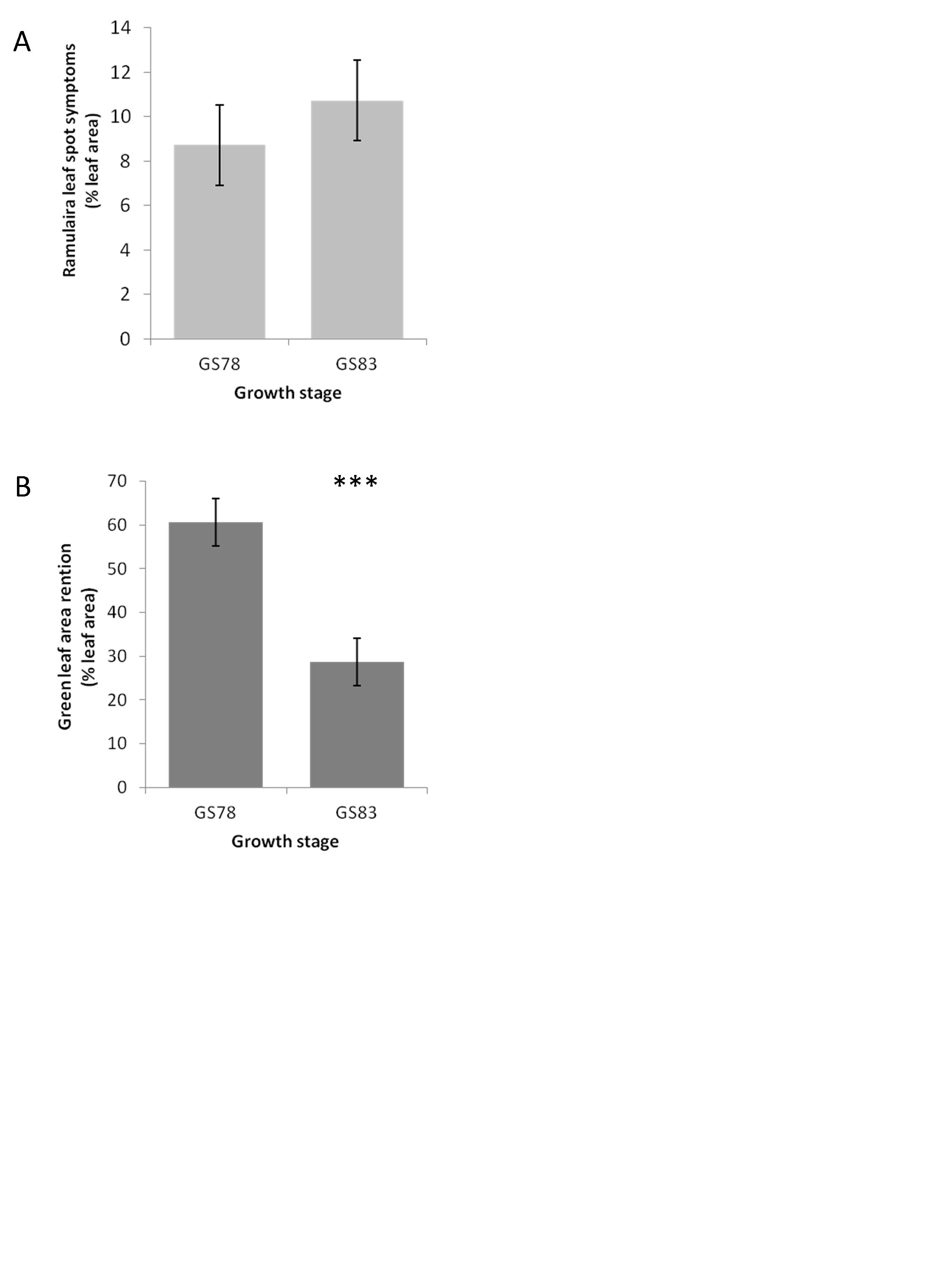

Supplement: Additional file 8: Figure S2. — Data from naturally infected Ramularia leaf spot spring barley field trials. A. Ramularia leaf spot levels on cv. Concerto at GS78 and GS83 (% of the total leaf area). B. Green leaf area retention of Ramularia leaf spot infected samples at GS78 and BS83. (TIF 902 kb) [file 12864_2016_2928_MOESM8_ESM.tif]

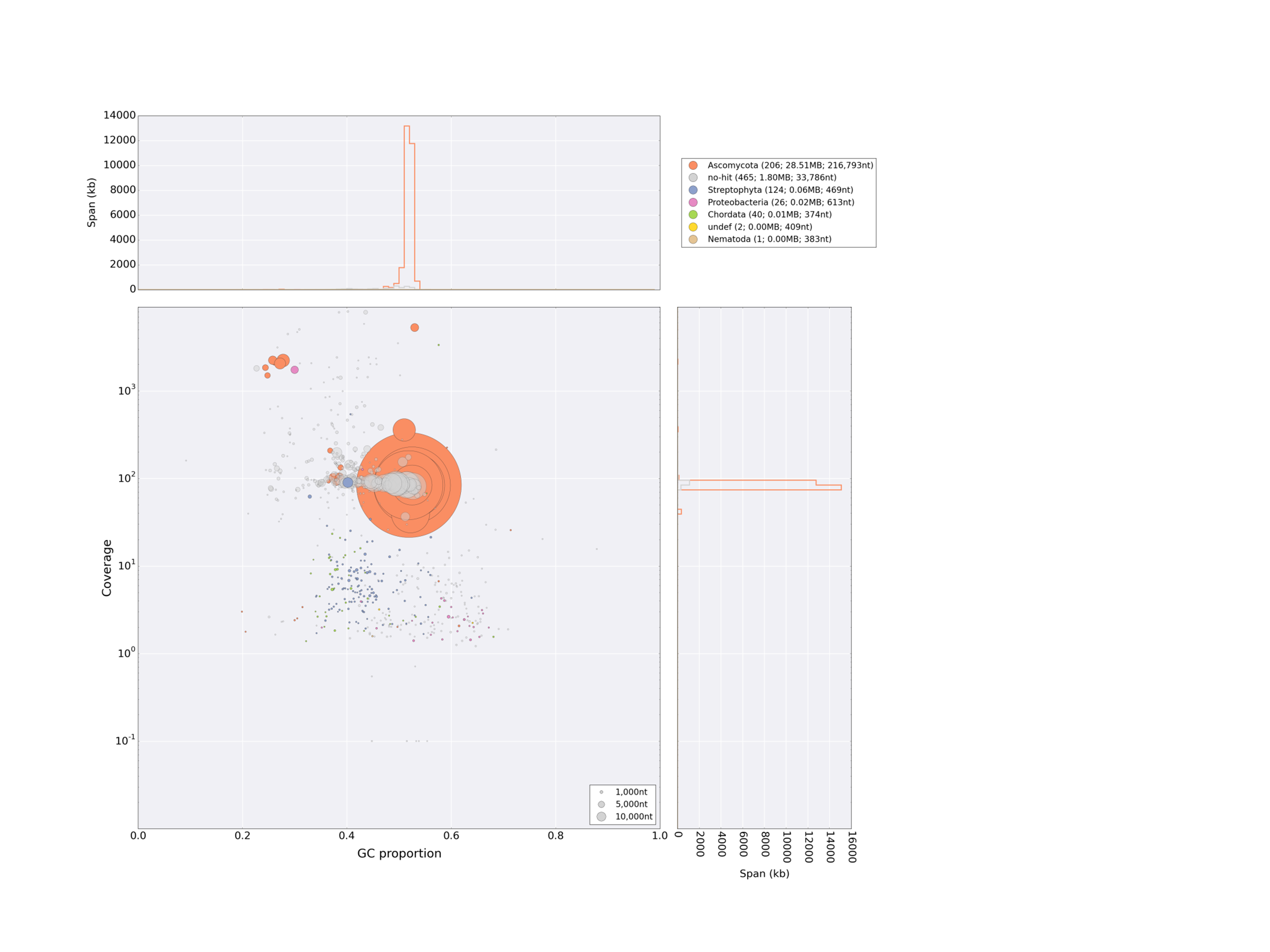

Supplement: Additional file 11: Figure S3. — Taxon annotated GC coverage plot of Ramularia collo-cygni genome assembly. (TIF 1542 kb) [file 12864_2016_2928_MOESM11_ESM.tif]
